# Mining KEAP1-Targeted Antioxidant Peptides from *Porphyra umbilicalis* Phycobiliproteins: In Silico Prioritization, Radical Scavenging, and Cellular Cytoprotection

**DOI:** 10.3390/foods15193488

**Published:** 2026-09-29

**Authors:** Fujia Yang, Wenting Jiang, Kun Qiao, Tingting Zhuo, Honglin Chen, Yingmei Zhang, Zhiyu Liu, Ronglong Jiang, Yijuan Han, Songbiao Chen

**Affiliations:** 1 Marine and Agricultural Biotechnology Laboratory, Fuzhou Institute of Oceanography, College of Geography and Oceanography, Minjiang University, 200 Xiyuangong Road, Shangjie Town, Minhou County, Fuzhou 350108, Chinayijuan.han@mju.edu.cn (Y.H.); 2 Key Laboratory of Cultivation and High-Value Utilization of Marine Organisms in Fujian Province, Fisheries Research Institute of Fujian, Xiamen 361013, China; 3 College of Biological Science and Engineering, Fuzhou University, Fuzhou 350108, China; 4 College of Ocean Food and Biological Engineering, Jimei University, Xiamen 361021, China; 5 Fujian Changheng Food Co., Ltd., Fuzhou 350500, China

**Keywords:** antioxidant peptides, KEAP1, virtual screening, molecular dynamics, cytoprotection

## Abstract

Phycobiliproteins from the red alga *Porphyra umbilicalis* are rich precursors of bioactive peptides, but targeted identification of high-potency sequences remains challenging. To address this, we combined an in silico screening pipeline with experimental validation to discover novel antioxidant peptides. Virtual enzymatic hydrolysis generated 908 candidate sequences, which were filtered down to 12 peptides based on molecular weight, toxicity, hydrophilicity, and bioactivity predictions. Molecular docking and molecular dynamics simulations further identified four candidate peptides (FK, FKP, FRS, and RRF) that were computationally predicted to bind stably within the KEAP1 Kelch domain. Chemical assays confirmed that all four synthesized peptides exhibited dose-dependent DPPH and ABTS radical-scavenging activities, along with Fe^2+^-chelating capacity and ferric reducing antioxidant power. In H_2_O_2_-stimulated Caco-2 cells, peptide pretreatment protected against cellular oxidative stress by restoring endogenous antioxidant enzyme activities, increasing total antioxidant capacity, and reducing lactate dehydrogenase leakage. Among these, RRF displayed the strongest protective efficacy. Overall, this study presents a practical workflow for mining marine-derived peptides and highlights four candidate peptides whose potential as functional food ingredients warrants further validation in actual *P. umbilicalis* hydrolysates.

## 1. Introduction

Uncontrolled accumulation of reactive oxygen species (ROS) disrupts cellular redox homeostasis, leading to oxidative stress and subsequent tissue damage [1]. This pathological state contributes to the development of major chronic conditions, including cardiovascular diseases, diabetes, neurodegenerative disorders, and cancer [2]. While synthetic antioxidants carry safety concerns, food-derived antioxidant peptides offer a natural, biocompatible alternative with diverse biological functions [3].

Marine organisms, particularly edible seaweeds, are exceptional sources of bioactive peptides due to their rich protein content and unique amino acid profiles [4]. Among these, *Porphyra* species represent economically valuable red algae containing 20% to 40% protein by dry weight. Phycobiliproteins constitute a main functional protein fraction in *Porphyra* and display marked antioxidant, anti-inflammatory, and immunomodulatory activities [5]. Traditionally, discovering antioxidant peptides from macroalgal proteins relies on physical enzymatic hydrolysis paired with activity-guided fractionations. These classical workflows, however, suffer from low throughput and risk losing highly active sequences during repetitive purification steps [6].

To overcome these limitations, in silico screening frameworks have transformed bioactive peptide discovery [7]. Combining virtual enzymatic hydrolysis, physicochemical filtering, molecular docking, and molecular dynamics (MD) simulations allows systematic evaluation of large sequence libraries before physical synthesis [8]. Specifically, molecular docking against the KEAP1 Kelch domain provides a mechanistically grounded approach to predict how peptide interactions with key endogenous antioxidant defense targets, offering clear rationales for candidate selection [9]. KEAP1 (Kelch-like ECH-associated protein 1) acts as a substrate adaptor for a CUL3-based ubiquitin ligase complex, and its Kelch domain recognizes the ETGE and DLG motifs of NRF2 (nuclear factor erythroid 2-related factor 2) Under basal conditions, this interaction promotes NRF2 ubiquitination and proteasomal degradation. Compounds or peptides that competitively interfere with the Kelch–NRF2 protein–protein interaction may stabilize NRF2, promote its nuclear accumulation, and induce antioxidant-response-element (ARE)-dependent genes such as HMOX1, NQO1, GCLC, and GCLM [10,11].

In this work, we applied a hybrid in silico and experimental workflow to discover and characterize antioxidant peptides from *P.* phycobiliproteins. Virtual cleavage generated initial peptide pools, which were filtered sequentially by molecular weight, predicted toxicity, hydrophilicity, and bioactivity scores. Top candidates were computationally fitted to the KEAP1 Kelch domain and verified through MD simulations to assess binding stability. Following computational prioritization, candidate peptides were synthesized to evaluate their chemical radical-scavenging capacities (DPPH, ABTS, Fe^2+^-chelation, and FRAP). Finally, we validated their cytoprotective effects in H_2_O_2_-damaged Caco-2 human intestinal cells by examining intracellular antioxidant enzyme recovery and membrane integrity markers. This study provides a targeted framework for mining high-value marine functional ingredients and elucidates the antioxidant potential of *P. umbilicalis* hydrolysates at both molecular and cellular levels.

## 2. Materials and Methods

### 2.1. Materials

Phycobiliprotein sequence data for *P. umbilicalis* (UniProt ID: J7F8E6) were retrieved from the UniProt database. Synthetic peptides FK, FKP, FRS, and RRF (purity ≥ 95%) were custom-synthesized by GL Biochem Ltd. (Shanghai, China) using Fmoc solid-phase peptide synthesis. Human intestinal epithelial cells (Caco-2) were purchased from Cell Bank of the Chinese Academy of Sciences (Shanghai, China). All chemical reagents and solvents were of analytical grade and purchased from Macklin Biochemical Co., Ltd. (Shanghai, China).

### 2.2. Sequence Acquisition and Virtual Enzymatic Hydrolysis

In silico enzymatic hydrolysis of the *P. umbilicalis* phycobiliprotein sequence (UniProt accession: J7F8E6; https://www.uniprot.org/uniprotkb/J7F8E6/entry; accessed on 18 May 2026) was performed using the PeptideCutter tool on the ExPASy server (https://web.expasy.org/peptide_cutter/, accessed on 18 May 2026). The full-length sequence was used without additional sequence boundaries, and cleavage was simulated using the “all available enzymes and chemicals” mode. Fragment sequences were deduplicated by removing identical entries, and single amino acid residues were excluded; the remaining unique fragments were compiled into an initial peptide library of 908 sequences [6,12,13].

### 2.3. In Silico Screening of Antioxidant Peptides

#### 2.3.1. Molecular Weight Filtering

Molecular weights were calculated using the ExPASy ProtParam tool (https://web.expasy.org/compute_pi/; accessed on 20 May 2026). Only peptides with molecular weights under 1000 Da were selected for subsequent screening [14].

#### 2.3.2. Toxicity Prediction

Potential peptide toxicity was evaluated using the ToxinPred server (https://webs.iiitd.edu.in/raghava/toxinpred/; accessed on 20 May 2026). Non-toxic sequences were retained for downstream analysis [15].

#### 2.3.3. Hydrophilicity Analysis

Peptide hydrophilicity was assessed via the ToxinPred portal (https://webs.iiitd.edu.in/raghava/toxinpred/; accessed on 20 May 2026). Peptides with hydrophilicity values above 0 were selected to ensure adequate aqueous solubility for potential food applications [16].

#### 2.3.4. Bioactivity Prediction

Bioactivity potential was predicted using the PeptideRanker server (https://peptide.ucd.ie/peptideranker/; accessed on 20 May 2026). Sequences scoring above 0.5 were shortlisted for molecular docking.

### 2.4. Molecular Docking

Molecular docking was conducted using Discovery Studio 2019 (Accelrys Software Inc., San Diego, CA, USA). Candidate peptide structures were drawn in ChemDraw 2020 and optimized using the “Prepare Ligands” module in Discovery Studio. The crystal structure of the human KEAP1 Kelch domain bound to an NRF2 peptide (PDB ID: 2FLU) was downloaded from the RCSB Protein Data Bank. The receptor was prepared via the “Prepare Protein” module by removing water molecules and co-crystallized ligands, adding hydrogen atoms, repairing missing residues, and performing CHARMm force field minimization. The active site was defined by the native NRF2-binding region in the KEAP1 Kelch domain. Docking poses were ranked by −CDOCKER interaction energy, and conformations exhibiting low binding energy and favorable interaction profiles were selected for further study [17].

### 2.5. Molecular Dynamics Simulation

Molecular dynamics (MD) simulations were executed in GROMACS using the OPLS-AA force field. The peptide–KEAP1 complex was centered in a cubic box with a 1.0 nm minimum margin and solvated with TIP3P water molecules. System charges were neutralized with Na^+^ and Cl^−^ ions to reach a physiological ionic concentration of 0.15 mol/L. Energy minimization was performed using the steepest descent algorithm for up to 50,000 steps. Systems then underwent NVT equilibration (100 ps, 300 K) and NPT equilibration (1 ns, 1 bar). Production MD simulations ran for 300 ns with a 2 fs time step, saving trajectory frames every 10 ps. Root mean square deviation (RMSD), radius of gyration (Rg), potential energy, and hydrogen bond counts were calculated to assess complex stability [18].

### 2.6. Peptide Synthesis and Purification

Selected peptides were synthesized on Rink amide MBHA resin using standard Fmoc solid-phase chemistry. The synthesized peptides were cleaved with a trifluoroacetic acid (TFA) cleavage cocktail and precipitated with cold diethyl ether. Crude peptides were purified by RP-HPLC to achieve purity greater than 95%, with target masses verified by ESI-MS.

### 2.7. In Vitro Antioxidant Activity Assays

#### 2.7.1. DPPH Radical Scavenging Assay

DPPH radical scavenging capacity was determined with slight modifications to established procedures. Briefly, 100 μL of peptide solution (0.625–20 mg/mL) was mixed with 100 μL of 0.2 mM ethanolic DPPH solution and incubated in the dark for 30 min. Reduced glutathione (GSH) was used as the positive control. Absorbance was recorded at 517 nm on a microplate reader (SpectraMax M5, Molecular Devices, Sunnyvale, CA, USA) [19].

#### 2.7.2. ABTS Radical Scavenging Assay

ABTS radical cations were generated by reacting 7 mM ABTS solution with 2.45 mM potassium persulfate in the dark at room temperature for 12–16 h. The stock radical solution was diluted with PBS to obtain an ABTS working solution with an absorbance of 0.70 ± 0.02 at 734 nm. Briefly, 100 μL of peptide solution (0.625–20 mg/mL) was mixed with 100 μL of ABTS working solution. Reduced glutathione (GSH) was used as the positive control. The mixture was incubated at room temperature for 6 min, and the absorbance was recorded at 734 nm [19].

#### 2.7.3. Ferrous Ion Chelating Assay

Ferrous ion chelating activity was measured according to a reported method with minor modifications. Briefly, 100 μL of peptide solution (0.625–20 mg/mL) was mixed with 50 μL of 2 mM FeCl_2_ solution. EDTA was used as the positive control. Subsequently, 50 μL of 5 mM ferrozine solution was added to initiate the reaction. The mixture was incubated at room temperature for 10 min, and the absorbance was determined at 562 nm [20].

#### 2.7.4. FRAP Assay

Ferric reducing antioxidant power (FRAP) assay was performed with minor modifications. Briefly, 100 μL of peptide solution (0.625–20 mg/mL) was mixed with 100 μL freshly prepared FRAP reagent. FRAP values were expressed as micromole Trolox equivalents per gram of peptide. The mixture was incubated at 37 °C for 30 min, and the absorbance was recorded at 593 nm [20].

#### 2.7.5. Dose–Response Analysis

Percentage inhibition in the DPPH, ABTS, and Fe^2+^-chelating assays was plotted against peptide concentration (0.625–20 mg/mL), and dose–response curves were fitted via nonlinear regression using a four-parameter logistic equation. The half-maximal inhibitory concentration (IC_50_) values for the DPPH and ABTS assays and the half-maximal effective concentration (EC_50_) values for the Fe^2+^-chelating assay were calculated from the fitted curves. Reduced glutathione (GSH) and EDTA served as positive controls in the radical-scavenging and Fe^2+^-chelating assays, respectively.

### 2.8. Cellular Antioxidant Assays in Caco-2 Cells

Caco-2 cells were seeded in 96-well plates and grown to 80–90% confluence. For cytotoxicity screening, cells were treated with increasing final concentrations of RRF (0.0625–1 mg/mL) for 24 h, and cell viability was measured using CCK-8 (Beyotime Biotechnology, Shanghai, China) to define a safe working concentration range. For the oxidative-stress experiments, cells were pretreated with GSH (positive control) or the peptides at low (0.05 mg/mL), medium (0.15 mg/mL), and high (0.25 mg/mL) concentrations for 24 h and subsequently exposed to 1 mM H_2_O_2_ for 4 h, after which cell viability was determined. For the biochemical assays, cells pretreated with 0.25 mg/mL peptide were exposed to 1 mM H_2_O_2_ for 4 h. Culture media were collected to measure lactate dehydrogenase (LDH) leakage [21]. Cells were washed twice with ice-cold PBS, lysed with cell lysis buffer, and centrifuged at 10,000× *g* for 10 min at 4 °C. Supernatants were collected for biochemical analyses. Total protein concentration was determined using a BCA protein assay kit (Nanjing Jiancheng Bioengineering Institute, Nanjing, China) to normalize cellular enzyme activities.

Intracellular superoxide dismutase (SOD) activity, glutathione peroxidase (GSH-Px) activity, total antioxidant capacity (T-AOC), and supernatant LDH release were measured using dedicated assay kits (Solarbio, Beijing, China) strictly following the manufacturer’s guidelines.

### 2.9. Statistical Analysis

All experiments were performed in triplicate, and data are presented as mean ± standard deviation (SD). Statistical significance was evaluated using one-way analysis of variance (ANOVA) followed by Duncan’s multiple range test in SPSS 26.0 software (IBM Corp., Armonk, NY, USA). Differences were considered statistically significant at *p* < 0.05.

## 3. Results and Discussion

### 3.1. Integrated Screening Strategy for Antioxidant Peptides

**Figure 1:**
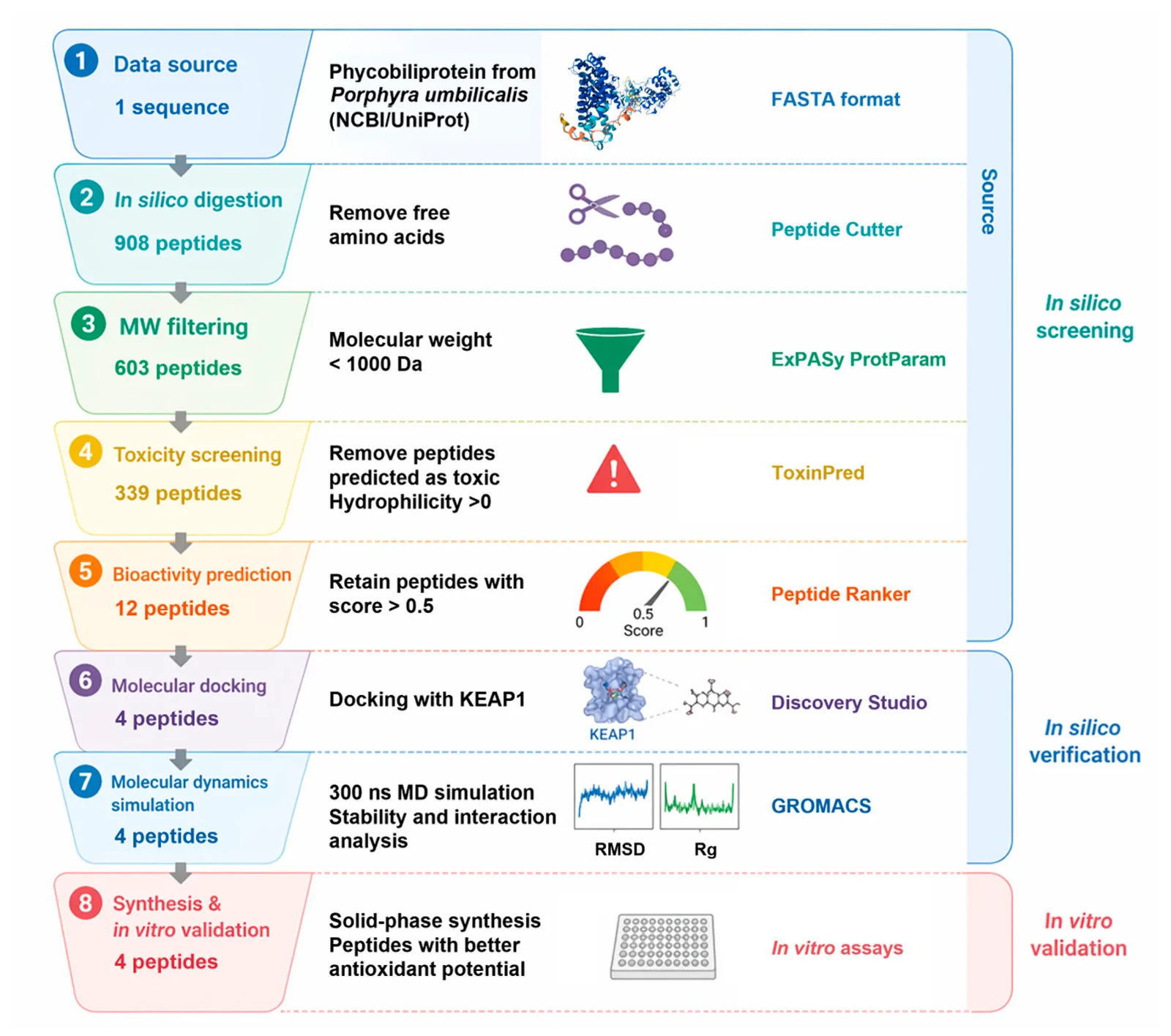
Graphical workflow of the integrated screening strategy for antioxidant peptides derived from phycobiliproteins of *Porphyra umbilicalis*.

To efficiently mine potent antioxidant sequences from *P. umbilicalis* phycobiliproteins, we implemented a multi-stage computational and experimental workflow (Figure 1). Initial virtual enzymatic cleavage of protein sequences retrieved from UniProt generated a broad peptide library [8,22]. We then applied sequential filters targeting molecular weight, safety, solubility, and predicted bioactivity to rapidly eliminate non-target sequences. Promising candidates were docked into the active pocket of the KEAP1 Kelch domain, and their binding stability was evaluated under dynamic conditions through MD simulations. Finally, top-performing peptides were synthesized for empirical validation using chemical and cellular antioxidant assays.

### 3.2. Screening Results and Physicochemical Characteristics of Candidate Peptides

**Figure 2:**
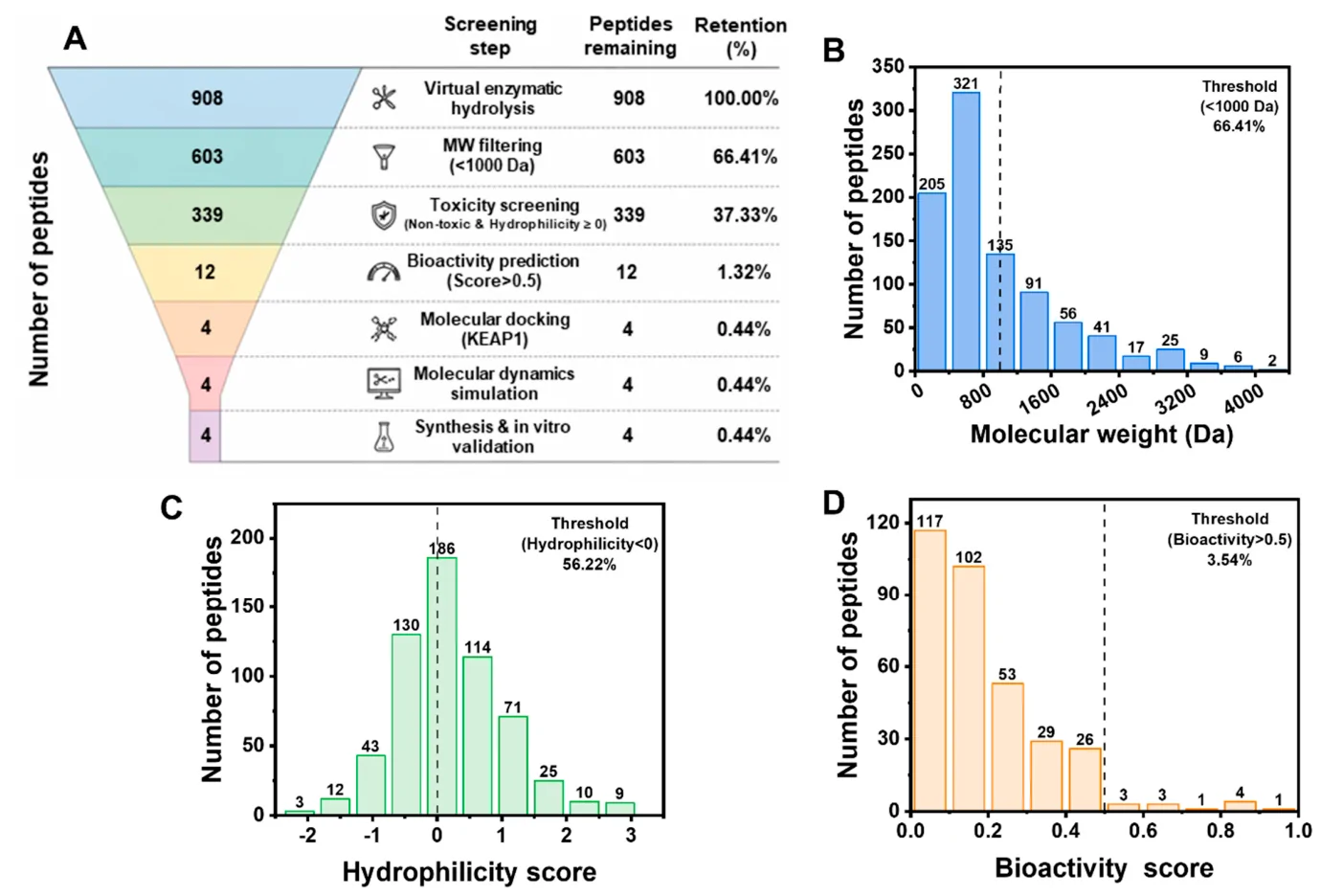
Statistical analysis of screening results and physicochemical properties of candidate peptides. (**A**) Number of peptides remaining after each screening step; (**B**) molecular weight distribution; (**C**) hydrophilicity value distribution; (**D**) bioactivity score distribution.

The stepwise reduction in the peptide pool demonstrates the high selectivity of this multi-tier pipeline (Figure 2A). Virtual hydrolysis of phycobiliproteins initially yielded 908 unique sequences. Restricting molecular weight to under 1000 Da retained 603 peptides, representing 66.41% of the initial library. Subsequent toxicity and hydrophilicity filtering excluded poorly soluble or toxic sequences, leaving 339 candidates (37.33%). Bioactivity prediction via PeptideRanker with a threshold above 0.5 narrowed this group to 12 peptides (1.32%). Ultimately, molecular docking against KEAP1 prioritized four candidate peptides for MD simulations and experimental validation, accounting for 0.44% of the original pool. This efficient funneling highlights how targeted computational screening can enrich highly active functional peptides from vast sequence spaces [23].

Profiling the physical properties of the generated peptides confirmed their structural suitability for biological applications. The majority of sequences concentrated below 800 Da, with 400 to 800 Da oligopeptides representing the primary population (Figure 2B). Low molecular weight is crucial for bioactivity, as smaller fragments navigate biological barriers more effectively and interact more readily with radical species or receptor sites. Hydrophilicity distribution among the 603 size-filtered peptides displayed an approximately normal curve, comprising 337 hydrophilic and 266 hydrophobic sequences (Figure 2C). This balanced amphiphilic character facilitates aqueous solubility in functional food matrices while retaining sufficient lipophilicity to interact with membrane lipids and cell surfaces. PeptideRanker bioactivity scores were largely clustered below 0.5, with only a select fraction achieving high scores (Figure 2D). This selective distribution confirmed that the screening pipeline effectively isolated high-potential sequences, laying a solid foundation for subsequent KEAP1 docking and bioactivity evaluations.

### 3.3. Molecular Docking Analysis of Peptide–KEAP1 Interactions

**Figure 3:**
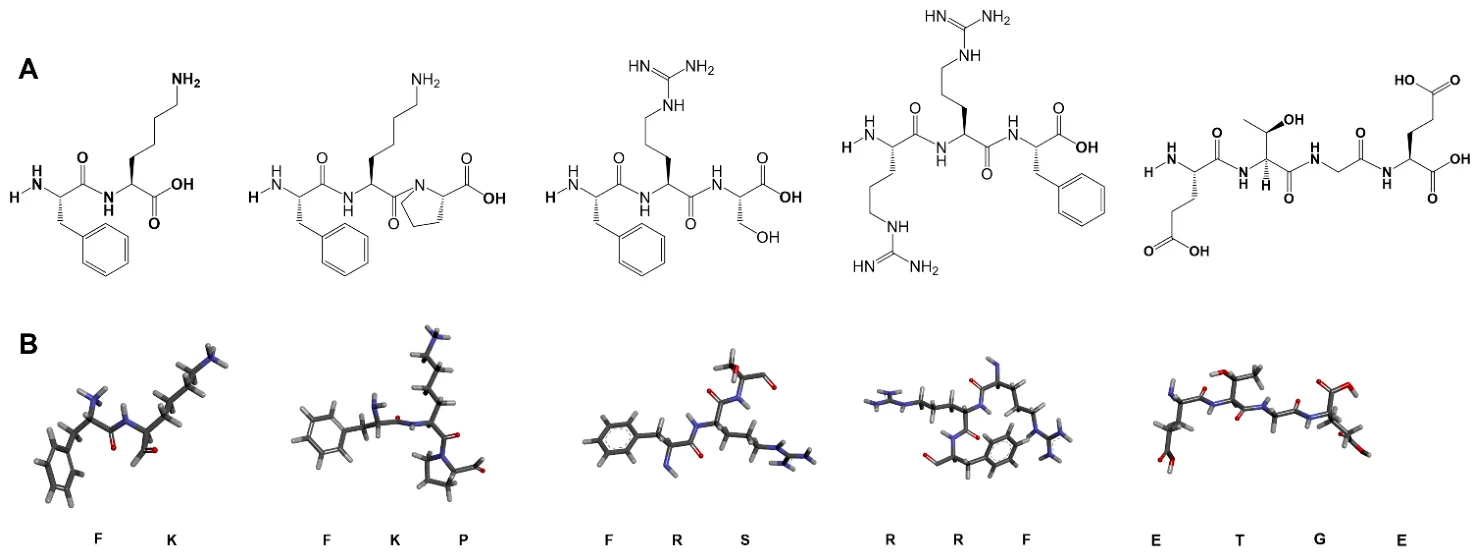
Chemical structures of candidate antioxidant peptides. (**A**) 2D structures; (**B**) 3D ball-and-stick models of FK, FKP, FRS, RRF and ETGE.

**Figure 4:**
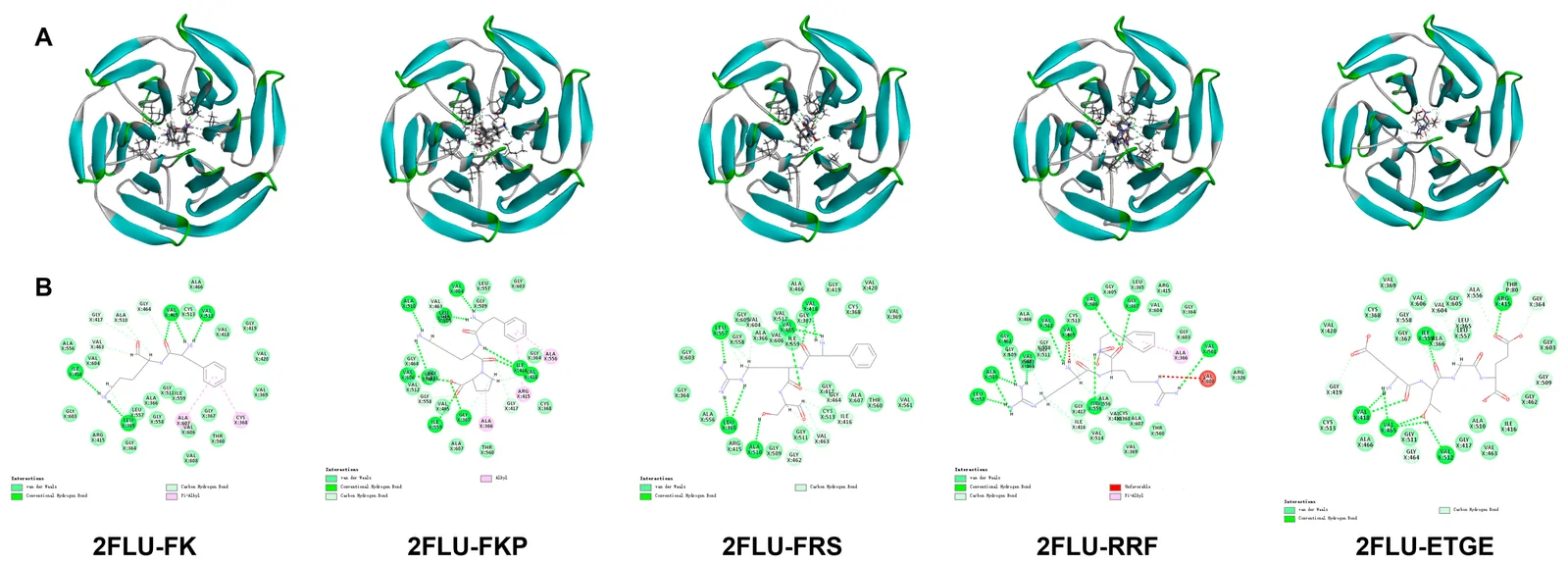
Molecular docking analysis of FK, FKP, FRS and RRF with KEAP1. (**A**) Docking conformations of peptides bound to KEAP1; (**B**) interaction force analysis between peptides and KEAP1.

To evaluate how candidate peptides engage key cellular antioxidant targets, we docked them into the KEAP1 Kelch domain, the primary pocket responsible for recognizing and sequestering NRF2 [10]. Four candidates (FK, FKP, FRS, and RRF) accommodated favorably within the KEAP1 binding cavity, exhibiting distinct structural features and binding modes (Figure 3A,B and Figure 4A). Within the phycobiliprotein ApcE sequence (UniProt J7F8E6), the four selected peptides correspond to the following regions: FKP (residues 133–135), FRS (residues 119–121 and 580–582), FK (residues 133–134, 510–511, 671–672, and 879–880), and RRF (residues 847–849).

Binding energetics and residue contact profiles varied among the four sequences (Table 1). As a structural benchmark, the native NRF2 ETGE motif exhibited a CDOCKER energy of −107.56 kcal/mol and an interaction energy of −87.1318 kcal/mol, forming 16 key contacts with residues such as Gly558, Ile559, Val604, and Arg415. FRS recorded the highest binding affinity, with a CDOCKER energy of −69.4502 kcal/mol and a CDOCKER interaction energy of −73.0310 kcal/mol. This strong binding stems from an extensive contact network involving Leu557, Val604, Val465, Val418, Gly464, Ile416, Val463, Ala510, and Leu365 in the KEAP1 cavity (Figure 4B). RRF also bound tightly, achieving a CDOCKER interaction energy of −68.2675 kcal/mol and forming the highest density of intermolecular interactions among all candidates. RRF established contacts across key pocket residues, including Leu557, Ala510, Gly462, Val463, Val512, Gly558, Val465, Val606, Gly367, Val561, Ile416, Ile559, and Val418. Meanwhile, FK and FKP maintained stable docked poses with interaction energies of −62.3381 and −65.3940 kcal/mol, respectively, engaging residues such as Ile416, Val463, Ala510, Val604, and neighboring sites.

Crucially, several core contact residues (Ile416, Val418, Val463, Ala510, and Val512) directly overlap with or neighbor the canonical NRF2-binding region within KEAP1. Occupying these critical hot spots suggests that the peptides may competitively interfere with the KEAP1–NRF2 interaction [24]. Specific side-chain features explain these computational observations. Abundant hydrophobic residues (Phe, Leu, Val, and Ile) across all four sequences promote hydrophobic packing within the non-polar KEAP1 cavity. Aromatic Phe residues further enhance van der Waals interactions and spatial stability, whereas the basic Arg residues in RRF introduce strong electrostatic interactions and hydrogen bonding, providing a structural rationale for its high contact density. Overall, these favorable molecular interactions underscore the ability of FK, FKP, FRS, and RRF to interfere with KEAP1 binding, although these findings remain predictive [25,26].

### 3.4. Molecular Dynamics Simulation of Peptide–KEAP1 Complexes

**Figure 5:**
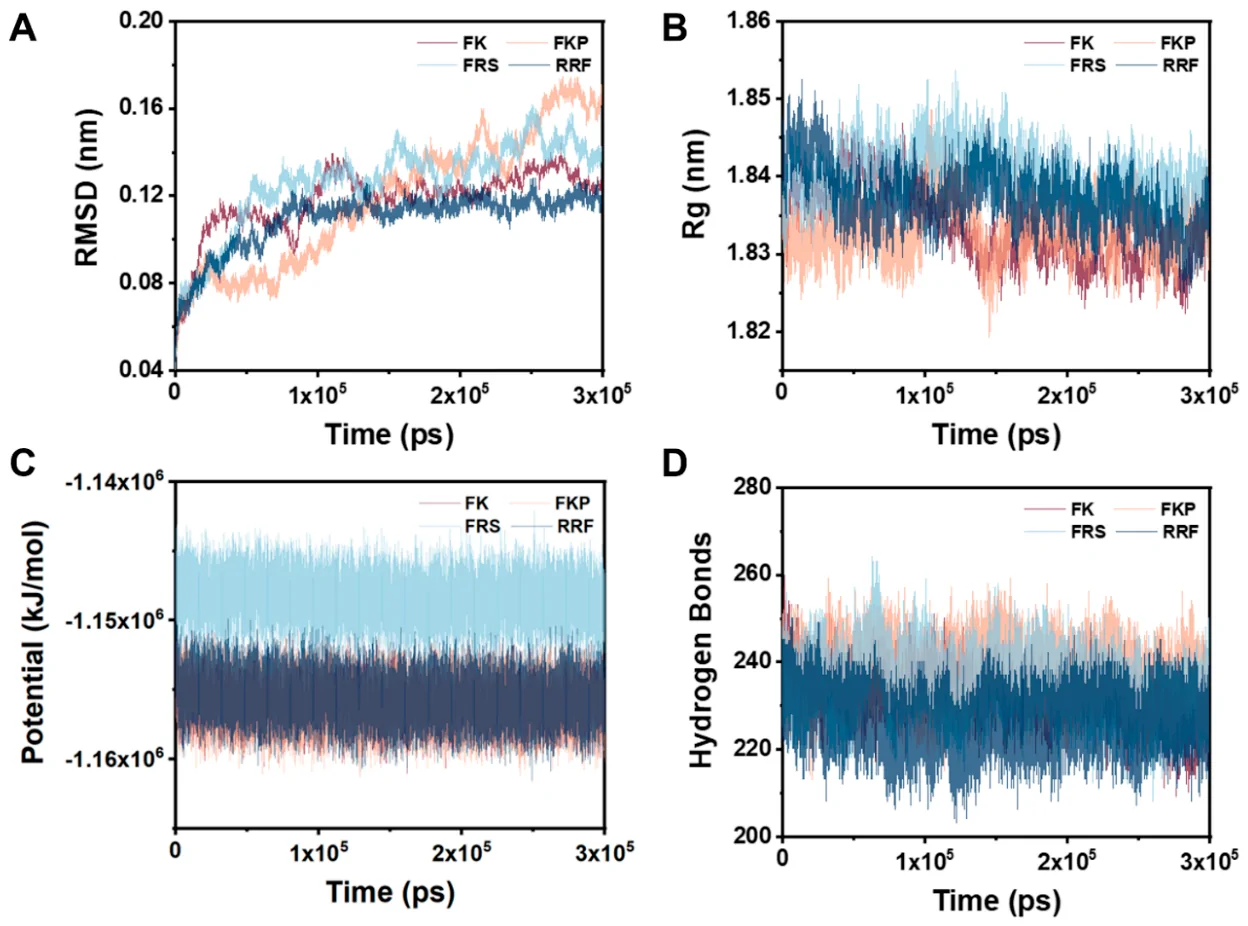
Molecular dynamics simulation results of peptide-KEAP1 complexes. (**A**) RMSD; (**B**) radius of gyration; (**C**) potential; (**D**) hydrogen bond numbers.

To evaluate the dynamic stability of the docked poses under simulated physiological conditions, we conducted 300 ns MD simulations for the FK, FKP, FRS, and RRF complexes with KEAP1. All four systems experienced an initial rise in RMSD during the first 100 ns, reflecting structural relaxation as the peptides optimized their orientations within the binding pocket (Figure 5A). Beyond 100 ns, the trajectories reached steady equilibrium. The RRF–KEAP1 system maintained the lowest RMSD fluctuations throughout the simulation, indicating exceptional conformational stability. In contrast, FKP–KEAP1 displayed greater structural flexibility during the final 100 ns [9].

Radius of gyration (Rg) metrics further confirmed that peptide binding preserved the global compactness of the KEAP1 Kelch domain (Figure 5B). Throughout the 300 ns run, Rg values across all four systems remained remarkably flat without sudden spikes or expansion, showing that peptide occupancy did not disturb the native fold of KEAP1. Among the tested candidates, FRS–KEAP1 and RRF–KEAP1 exhibited the narrowest Rg distributions, reflecting superior structural rigidity upon complexation.

Thermodynamic evaluations showed that potential energy profiles remained constant across all systems without energy drift or abrupt fluctuations, confirming system convergence and thermodynamic equilibrium (Figure 5C) [27]. Intermolecular hydrogen bond tracking underscored the persistent non-covalent network anchoring the peptides within the binding pocket (Figure 5D). Despite expected dynamic fluctuations caused by thermal movement, FKP–KEAP1 and FRS–KEAP1 maintained higher average hydrogen bond occupancies, strengthening their retention within the Kelch domain [11].

In summary, the 300 ns MD trajectories validate the dynamic stability and structural persistence of all four peptides within the KEAP1 binding pocket. Aligning with molecular docking predictions, FRS and RRF demonstrated superior dynamic stability and structural compactness. These computational findings suggest that these marine-derived peptides form stable interactions with KEAP1 in silico, providing a structural foundation for their antioxidant activities [28,29].

### 3.5. In Vitro Antioxidant Activities of Synthetic Peptides

All four candidate peptides (FK, FKP, FRS, and RRF) were obtained via Fmoc solid-phase synthesis with purities exceeding 95%. Electrospray ionization mass spectrometry (ESI-MS) confirmed that the observed molecular masses matched theoretical values (Supporting Information).

**Figure 6:**
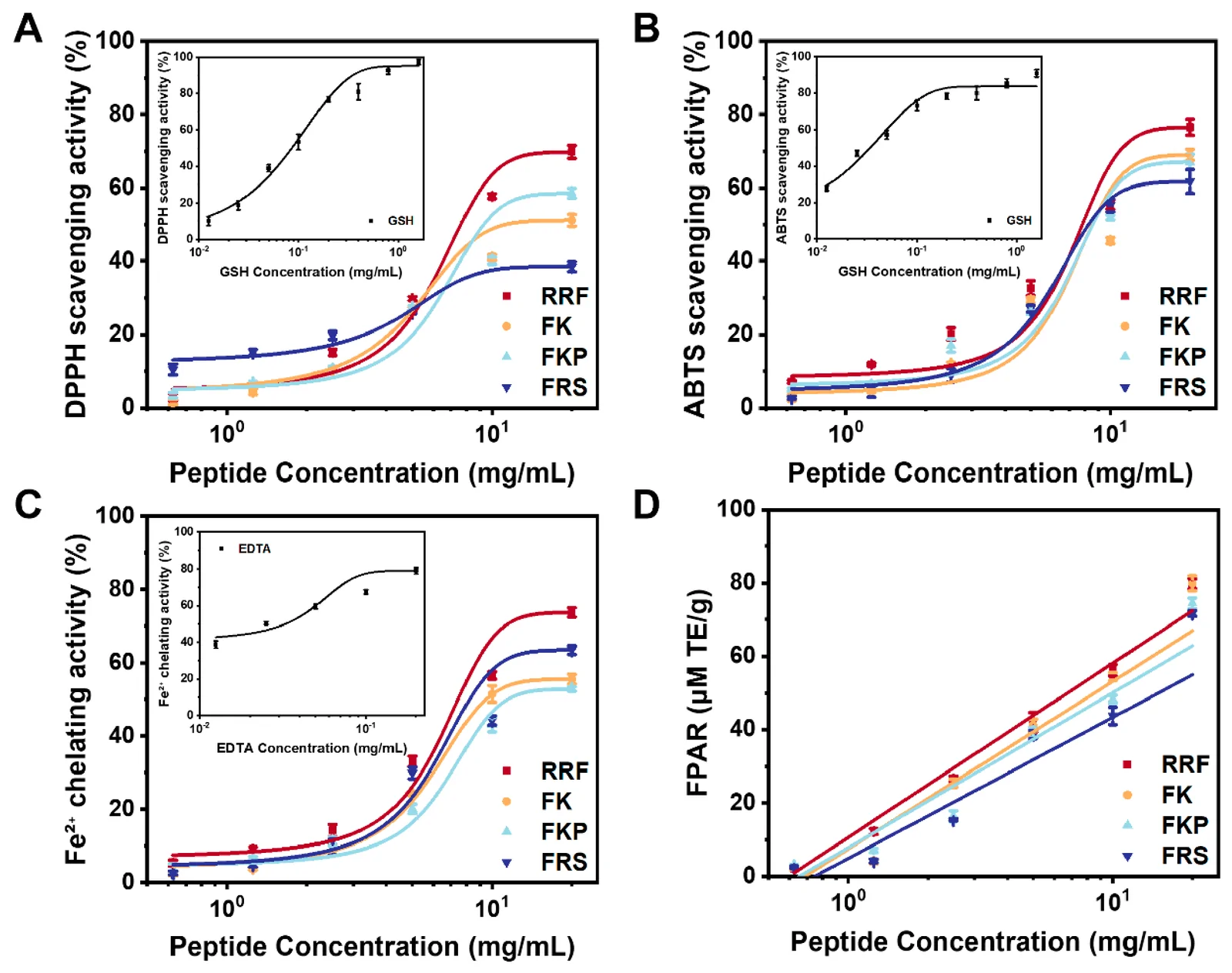
In vitro antioxidant activities of synthetic peptides. (**A**) DPPH radical scavenging activity; (**B**) ABTS radical scavenging activity; (**C**) Fe^2+^ chelating activity; (**D**) ferric reducing antioxidant power.

Chemical antioxidant profiling across the synthesized peptides demonstrated distinct radical-quenching and metal-chelating capacities. In the DPPH assay, all four peptides scavenged DPPH radicals in a concentration-dependent manner from 0.625 to 20 mg/mL (Figure 6A), following the order RRF > FKP > FK > FRS, with IC_50_ values of 8.31, 8.87, and 18.43 mg/mL for RRF, FKP, and FK, respectively. These values indicate that all four sequences can act as hydrogen atom or electron donors to neutralize lipophilic DPPH radicals [30]. RRF displayed the highest scavenging efficiency, which can be attributed to its two Arg residues. The guanidinium side chain of Arg promotes proton donation and stabilizes radical intermediates via resonance delocalization and electrostatic interactions. Additionally, N-terminal aromatic Phe residues across all sequences provide π-electrons that reinforce radical stabilization through resonance.

The peptides exhibited concentration-dependent scavenging of hydrophilic ABTS radical cations from 0.625 to 20 mg/mL (Figure 6B). RRF outperformed the other candidates, followed by FK, FRS, and FKP. The enhanced ABTS quenching compared with DPPH stems from the hydrophilic nature of ABTS cations, which interact more readily with short-chain peptides in aqueous systems [31]. Positively charged Arg side chains in RRF further facilitate electron transfer toward charged radical species, underscoring the vital role of basic residues in aqueous radical neutralization. Consistently, the ABTS IC_50_ values (6.29–8.63 mg/mL) were lower than the corresponding DPPH IC_50_ values (8.31–18.43 mg/mL), further confirming that the peptides quench the hydrophilic ABTS radical cation more efficiently than the lipophilic DPPH radical.

Transition metal sequestration was evaluated via ferrous ion (Fe^2+^) chelation, which increased with peptide concentration from 0.625 to 20 mg/mL (Figure 6C). RRF and FRS yielded the lowest Fe^2+^-chelating EC_50_ values (7.20 and 7.45 mg/mL, respectively), corroborating the superior chelating efficacy of these two sequences. Sequestering Fe^2+^ represents a critical indirect antioxidant mechanism, as it suppresses Fenton-type reactions that generate highly destructive hydroxyl radicals. The elevated chelating capacities of RRF and FRS are directly tied to their basic and polar amino acids (Arg and Ser), which supply electron-rich amino, hydroxyl, and carbonyl groups to form stable coordination complexes with iron ions [32].

Ferric reducing antioxidant power (FRAP) values increased with peptide concentration, ranging from 2.08 ± 0.39 to 79.77 ± 1.37 μmol TE/g across the tested range (Figure 6D). Because the FRAP assay operates strictly through a single-electron transfer (SET) mechanism under acidic conditions, the pronounced dose-dependent increase in reducing power indicates that all four peptides act as efficient electron donors, with sequence variations exerting a moderate influence on the magnitude of ferric reduction [33].

Dose–response analysis revealed concentration-dependent radical-scavenging and metal-chelating activities for all four peptides from 0.625 to 20 mg/mL (Figure 6A–D; Table 2). The DPPH IC_50_ values were 8.31 (RRF), 8.87 (FKP), and 18.43 (FK) mg/mL, whereas that for FRS could not be determined because its scavenging activity did not reach 50% within the tested range (38.5% at 20 mg/mL). The ABTS IC_50_ values were 6.29 (RRF), 7.20 (FK), 7.23 (FKP), and 8.63 (FRS) mg/mL, and the Fe^2+^-chelating EC_50_ values were 7.20 (RRF), 7.45 (FRS), 9.75 (FKP), and 10.42 (FK) mg/mL. As FRAP values are expressed as μmol TE/g rather than percentage inhibition and showed an approximate linear correlation with peptide concentration, no IC_50_/EC_50_ value was derived for this endpoint. Under identical conditions, the positive controls showed substantially higher potencies, with DPPH and ABTS IC_50_ values of 0.076 and 0.032 mg/mL for GSH and an Fe^2+^-chelating EC_50_ of 0.0252 mg/mL for EDTA. Notably, RRF consistently yielded the lowest IC_50_/EC_50_ values across all three dose–response assays, corroborating its superior activity at 5 mg/mL.

### 3.6. Protective Effects of Peptides Against H_2_O_2_-Induced Oxidative Stress in Caco-2 Cells

**Figure 7:**
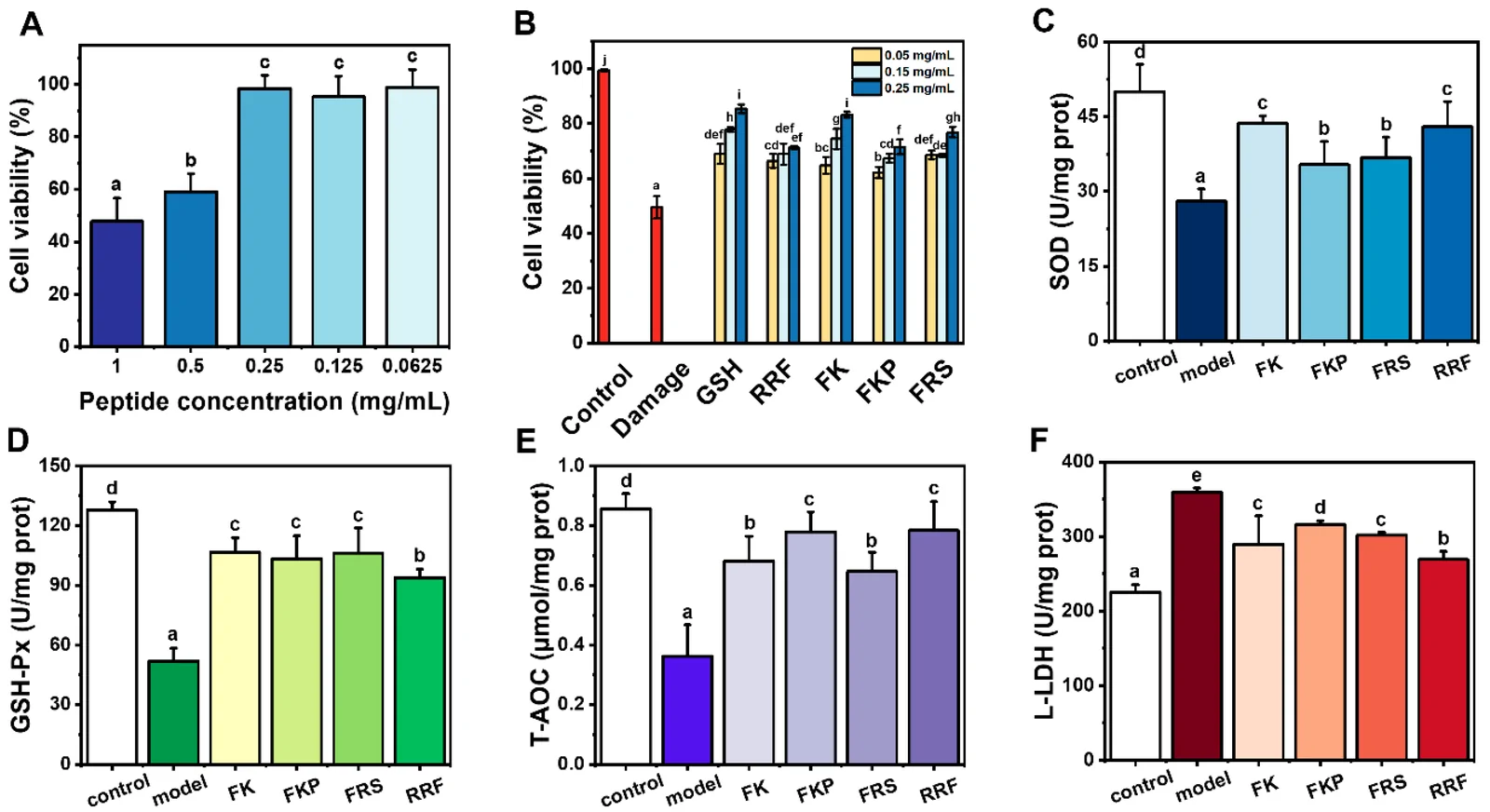
Protective effects of synthetic peptides against H_2_O_2_-induced oxidative stress in Caco-2 cells. (**A**) Cell viability of Caco-2 cells after 24 h treatment with increasing concentrations of RRF (0.0625–1 mg/mL); (**B**) cell viability of H_2_O_2_-damaged Caco-2 cells pretreated with the peptides at 0.05, 0.15, and 0.25 mg/mL; (**C**) superoxide dismutase (SOD) activity; (**D**) glutathione peroxidase (GSH-Px) activity; (**E**) total antioxidant capacity (T-AOC); (**F**) lactate dehydrogenase (LDH) release. Different lowercase letters indicate significant differences between groups (*p* < 0.05).

To define a safe working concentration range, the cytotoxicity of RRF—the most active peptide in the chemical assays—was first evaluated in Caco-2 cells. Treatment with RRF at 0.0625–0.25 mg/mL for 24 h did not significantly affect cell viability, whereas 0.5 and 1 mg/mL led to a significant decrease, indicating that concentrations up to 0.25 mg/mL were non-cytotoxic (Figure 7A). Accordingly, 0.25 mg/mL was selected as the highest working concentration for subsequent oxidative-stress experiments.

Exposure to 1 mM H_2_O_2_ for 4 h substantially reduced cell viability, whereas pretreatment with GSH or the four peptides at 0.05, 0.15, and 0.25 mg/mL restored viability in a concentration-dependent manner, with 0.25 mg/mL providing the strongest protection (Figure 7B). On this basis, 0.25 mg/mL was adopted for subsequent biochemical assays.

Challenging cells with 1 mM H_2_O_2_ severely suppressed superoxide dismutase (SOD) activity (*p* < 0.05), indicating a breakdown in primary enzymatic defense against superoxide radicals (Figure 7C). Peptide pretreatment remarkably restored SOD levels, reaching 43.67 ± 1.50, 35.35 ± 4.66, 36.72 ± 4.16, and 42.97 ± 5.07 U/mg protein for FK, FKP, FRS, and RRF, respectively. FK and RRF mounted the strongest recovery. Restoring SOD activity enables cells to rapidly dismutate excessive superoxide anions into hydrogen peroxide, mitigating initial ROS accumulation [34].

A corresponding protective response occurred in glutathione peroxidase (GSH-Px) activity (Figure 7D). While H_2_O_2_ treatment depressed GSH-Px to 51.92 ± 6.45 U/mg protein, peptide pretreatment effectively doubled enzyme levels across all groups (106.53 ± 7.38 U/mg protein for FK, 103.28 ± 11.55 for FKP, 106.12 ± 12.69 for FRS, and 93.83 ± 4.17 for RRF). Because GSH-Px catalyzes the reduction in lipid hydroperoxides and hydrogen peroxide, its upregulation confirms that the peptides enhance intracellular peroxide clearance and limit lipid peroxidation [35].

Reflecting this reinforced enzymatic defense, total antioxidant capacity (T-AOC) recovered substantially in peptide-pretreated cells (Figure 7E). Exposure to H_2_O_2_ depleted T-AOC to 0.36 ± 0.11 μmol/mg protein, whereas pretreatment restored T-AOC levels to 0.65–0.78 μmol/mg protein across all peptide groups, with FKP and RRF achieving the highest values (0.78 μmol/mg protein). This broad recovery in overall reducing potential helps re-establish cellular redox balance under oxidative stress.

Cellular integrity was further verified by measuring lactate dehydrogenase (LDH) leakage into culture media (Figure 7F). H_2_O_2_ challenge induced severe membrane destabilization, escalating LDH release to 358.92 ± 6.06 U/mg protein. Pretreatment with the peptides attenuated LDH leakage, with values dropping to 289.79 ± 37.95 (FK), 315.98 ± 5.07 (FKP), 301.90 ± 3.62 (FRS), and 269.06 ± 10.93 U/mg protein (RRF). RRF provided the most robust membrane protection, aligning with its high Fe^2+^ chelation and KEAP1 binding stability. Minimizing LDH release demonstrates that these peptides protect plasma membrane structural integrity against lipid oxidative damage [36].

In summary, peptide pretreatment protects Caco-2 intestinal cells against acute oxidative injury through a coordinated intracellular response. By restoring primary antioxidant enzyme activities (SOD and GSH-Px), boosting net cellular antioxidant capacity (T-AOC), and suppressing membrane leakage (LDH), these peptides assist in maintaining intracellular redox homeostasis. Integrating these cell-based outcomes with our molecular docking and dynamic simulations suggests that the cytoprotective efficacy of these peptides involves both direct radical scavenging or metal chelation and KEAP1-mediated modulation of endogenous antioxidant defenses.

### 3.7. Structure–Activity Relationship and Intermolecular Interaction Analysis

**Figure 8:**
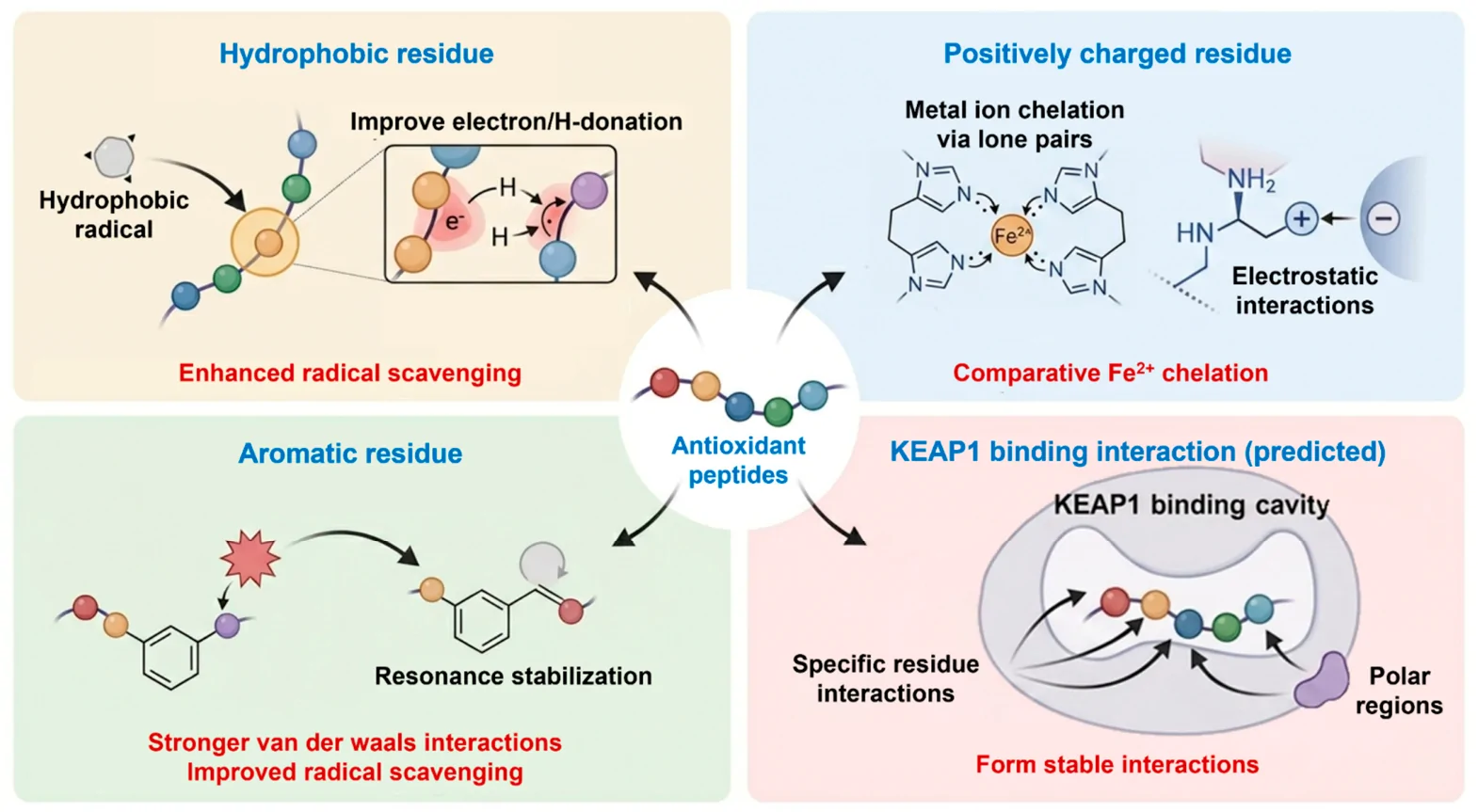
Schematic illustration of structure–activity relationship and intermolecular interaction mechanisms of antioxidant peptides.

The distinct antioxidant profiles among the identified peptides stem directly from their amino acid sequences, side-chain chemistry, and binding modes (Figure 8). Aromatic residues like Phe facilitate electron transfer and stabilize radical intermediates, while basic residues like Arg supply proton-donating sites, positive charges for electrostatic interactions, and coordination centers for metal chelation [37]. Consequently, the presence of two Arg residues in RRF explains its superior performance across DPPH, ABTS, and Fe^2+^-chelating assays [38].

At the molecular level, computational simulations confirmed that all four peptides settled stably into the KEAP1 Kelch domain [39]. Among them, RRF formed the most favorable binding conformations and persistent interaction stability, mirroring its high chemical reactivity. These molecular insights correlate directly with our cell-based observations. In H_2_O_2_-damaged Caco-2 cells, peptide pretreatment restored endogenous SOD and GSH-Px activities, elevated T-AOC, and suppressed LDH leakage, effectively preserving membrane integrity and intracellular redox balance [40].

Several limitations of this study should be acknowledged. First, the production of these four peptides during food-grade enzymatic digestion, their stability under gastrointestinal conditions, transport across the intestinal epithelium, and concentrations in the source material were not evaluated. Accordingly, the present results demonstrate the antioxidant potential of the individual synthetic peptides, which differs from the properties of a whole food hydrolysate, and conclusions regarding the value of *P. umbilicalis* hydrolysates as functional food ingredients should be considered preliminary. Second, based on the cleavage specificity of trypsin, FK, FRS, and RRF are susceptible to trypsin-catalyzed hydrolysis, whereas FKP contains a Lys-Pro bond that is resistant to trypsin, which is particularly relevant to the oral administration of these sequences. Third, the proposed KEAP1–NRF2 interaction is based solely on in silico predictions, and direct experimental evidence is required before any pathway-activating claim can be made. Future work should therefore validate the release, gastrointestinal stability, bioavailability, and KEAP1–NRF2-related activity of these peptides in genuine hydrolysates and in appropriate in vivo or cellular models.

## 4. Conclusions

This study establishes a targeted pipeline integrating in silico screening, molecular interactions, and multi-tier empirical validation to discover bioactive peptides from *P.* phycobiliproteins. Four novel candidates (FK, FKP, FRS, and RRF) were identified, all demonstrating robust DPPH and ABTS radical scavenging, Fe^2+^ chelation, and ferric reducing power. Computational docking and dynamic simulations predicted stable interactions between these peptides and the KEAP1 Kelch domain, offering a structural rationale for potential competitive KEAP1 binding that awaits experimental validation. In human Caco-2 intestinal cells, peptide pretreatment protected against oxidative damage by restoring SOD and GSH-Px enzyme activities, boosting total antioxidant capacity, and preventing membrane rupture. Among all candidates, the Arg-rich tripeptide RRF displayed the most potent overall efficacy across chemical, computational, and cellular evaluations. These findings demonstrate the utility of targeted computational screening for marine peptide discovery and highlight four candidate peptides whose potential as functional food ingredients should be further validated in actual *P. umbilicalis* hydrolysates, along with their gastrointestinal stability and bioavailability.

## Figures and Tables

**Table 1:** Docking energy and interacting residues of four peptides with KEAP1.

Peptide Sequences	CDOCKER Energy (kcal/mol)	CDOCKER Interaction Energy (kcal/mol)	Interactions	Residues
FK	−62.3381	−59.0883	12	Ile416, Val463, Gly417, Ala510, Gly464, Val465, Val512, Leu365
FKP	−59.4581	−65.3940	17	Ala510, Gly462, Val463, Leu365, Val604, Ile416, Val418, Gly417, Gly367, Ile559, Gly558, Val606
FRS	−69.4502	−73.0310	16	Leu557, Val604, Val465, Val418, Gly464, Ile416, Val463, Ala510, Leu365
RRF	−59.5232	−68.2675	21	Leu557, Ala510, Gly462, Val463, Val512, Gly558, Val465, Val606, Gly367, Val561, Ile416, Ile559, Val418
ETGE	−107.56	−87.1318	16	Gly558, Ile559, Val604, Leu365, Ala556, Arg415, Gly364, Gly509, Val512, Gly464, Val465, Val418, Gly419

**Table 2:** IC_50_ and EC_50_ values of the four peptides and positive controls in the chemical antioxidant assays.

Sample	DPPH IC_50_ (mg/mL)	ABTS IC_50_ (mg/mL)	Fe^2+^-Chelating EC_50_ (mg/mL)
FK	18.43	7.20	10.42
FKP	8.87	7.23	9.75
FRS	n.d.	8.63	7.45
RRF	8.31	6.29	7.20
GSH	0.076	0.0323	—
EDTA	—	—	0.0252

n.d. = not determinable: the DPPH scavenging activity of FRS did not reach 50% within the tested range.

## Data Availability

The original contributions presented in this study are included in the article/Supplementary Materials. Further inquiries can be directed to the corresponding authors.

## Supplementary Materials

The following supporting information can be downloaded at: https://www.mdpi.com/article/10.3390/foods15193488/s1, Figure S1: RP-HPLC chromatogram of peptide FK; Figure S2: ESI-MS spectrum of peptide FK; Figure S3. RP-HPLC chromatogram of peptide FKP; Figure S4. ESI-MS spectrum of peptide FKP; Figure S5. RP-HPLC chromatogram of peptide FRS; Figure S6. ESI-MS spectrum of peptide FRS; Figure S7. RP-HPLC chromatogram of peptide RRF; Figure S8. ESI-MS spectrum of peptide RRF.
