# Supplementary material for: Mining KEAP1-Targeted Antioxidant Peptides from Porphyra umbilicalis Phycobiliproteins: In Silico Prioritization, Radical Scavenging, and Cellular Cytoprotection
Source: Foods. 2026 Sep 29;15(19):3488. doi: 10.3390/foods15193488 (PMC13664560; doi:10.3390/foods15193488)
Supplement: Supplementary file 1 [file foods-15-03488-s001.zip › foods-4503099-supplementary.pdf]

Supporting Information

Mining KEAP1-targeted antioxidant peptides from *Porphyra umbilicalis* phycobiliproteins: *In silico* prioritization, radical scavenging, and cellular cytoprotection

Fujia Yang [a, b, # *], Wenting Jiang [a, c, #], Kun Qiao [b], Tingting Zhuo [a], Honglin Chen [a, c], Yingmei Zhang [a, d], Zhiyu Liu [b], Ronglong Jiang [e], Yijuan Han [a *], Songbiao Chen [a *]

[a] Marine and Agricultural Biotechnology Laboratory, Fuzhou Institute of Oceanography, College of Geography and Oceanography, Minjiang University, Fuzhou, 350108, P.R.China

[b] Key Laboratory of Cultivation and High-Value Utilization of Marine Organisms in Fujian Province, Fisheries Research Institute of Fujian, Xiamen 361013, P.R.China

[c] College of Chemical Engineering, College of Biological Science and Engineering, Fuzhou University, Fuzhou 350108, P.R.China

[d] College of Ocean Food and Biological Engineering, Jimei University, Xiamen 361021, China

[e] Fujian Changheng Food Co., Ltd, Fuzhou 350500, P.R.China

[#] Co-first author

Present address: 200 xiyuangong Road, Shangjie Town, Minhou County, Fuzhou City, Fujian Province, China, 350108

[*] Corresponding author: yangfujia@mju.edu.cn

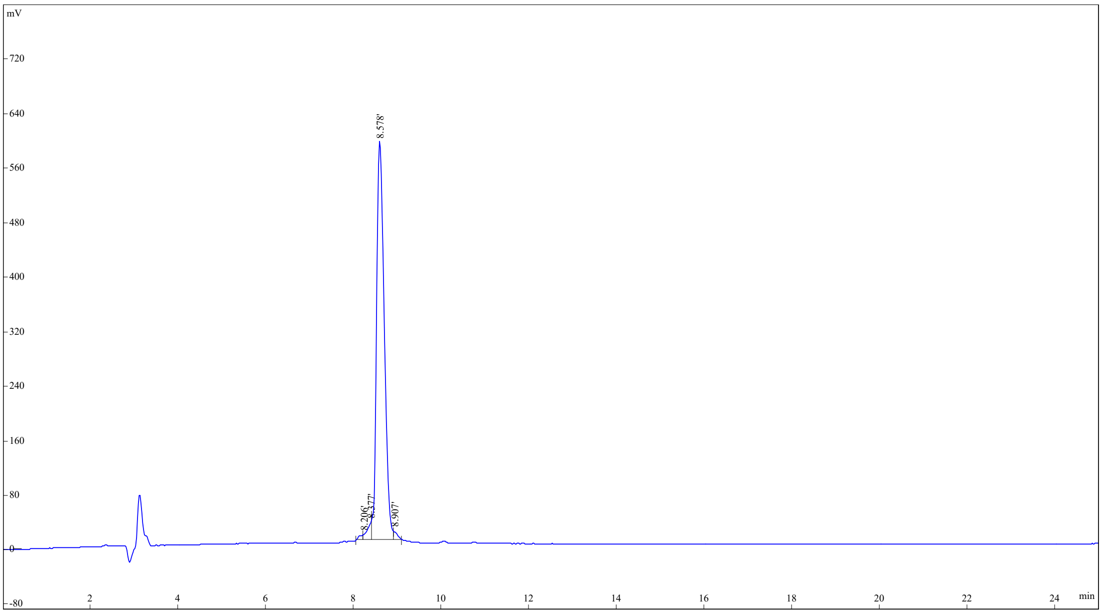

Figure S1. RP-HPLC chromatogram of peptide FK.

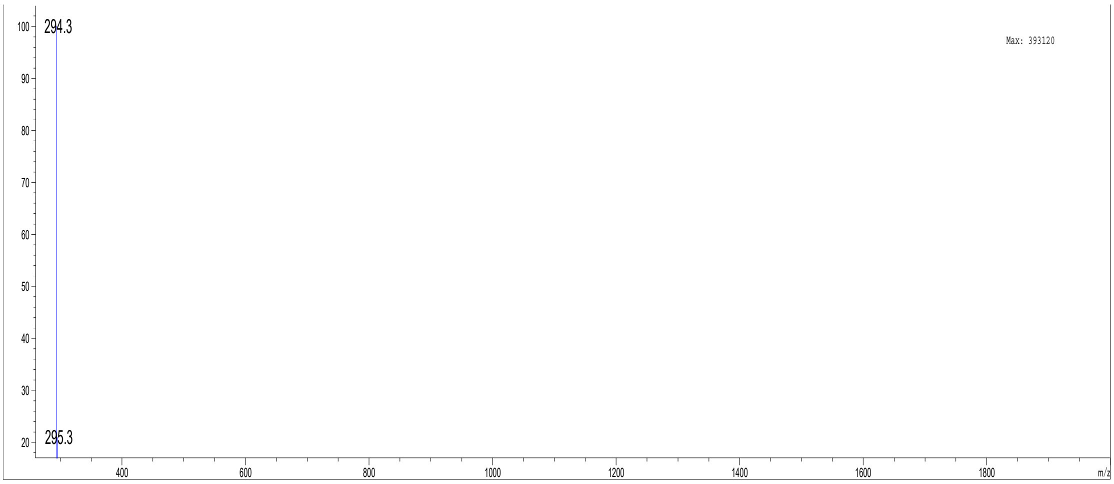

Figure S2. ESI-MS spectrum of peptide FK.

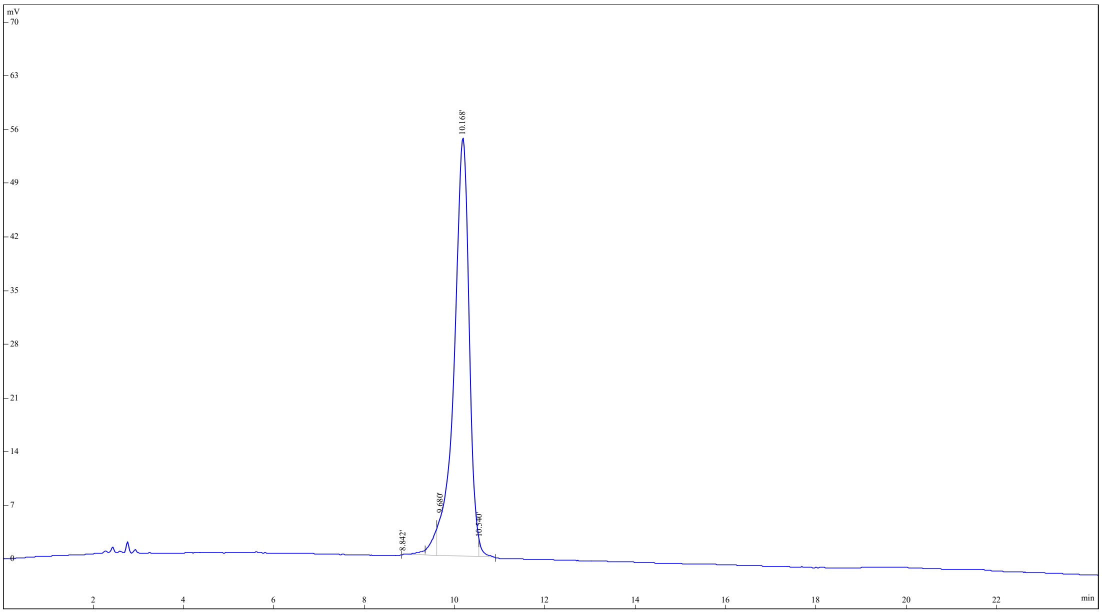

Figure S3. RP-HPLC chromatogram of peptide FKP.

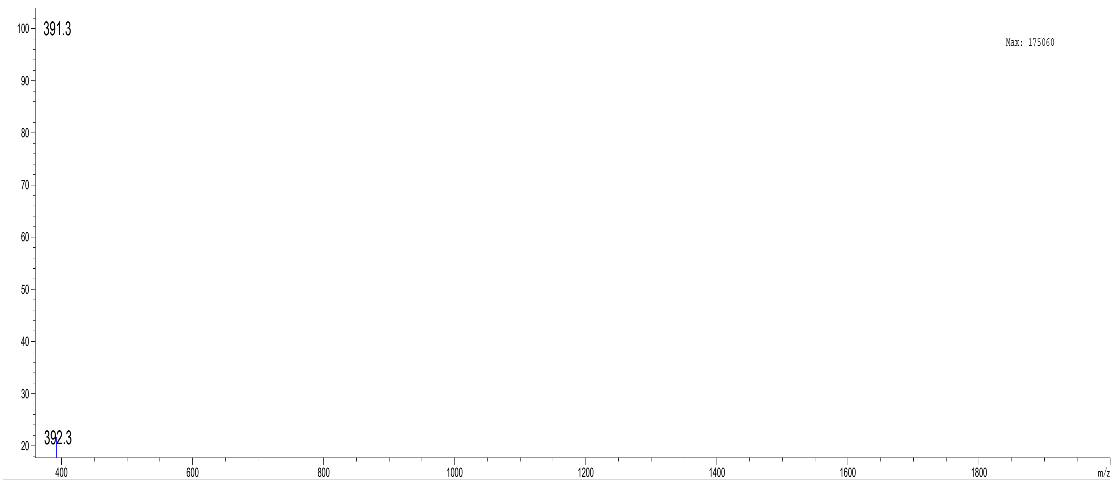

Figure S4. ESI-MS spectrum of peptide FKP.

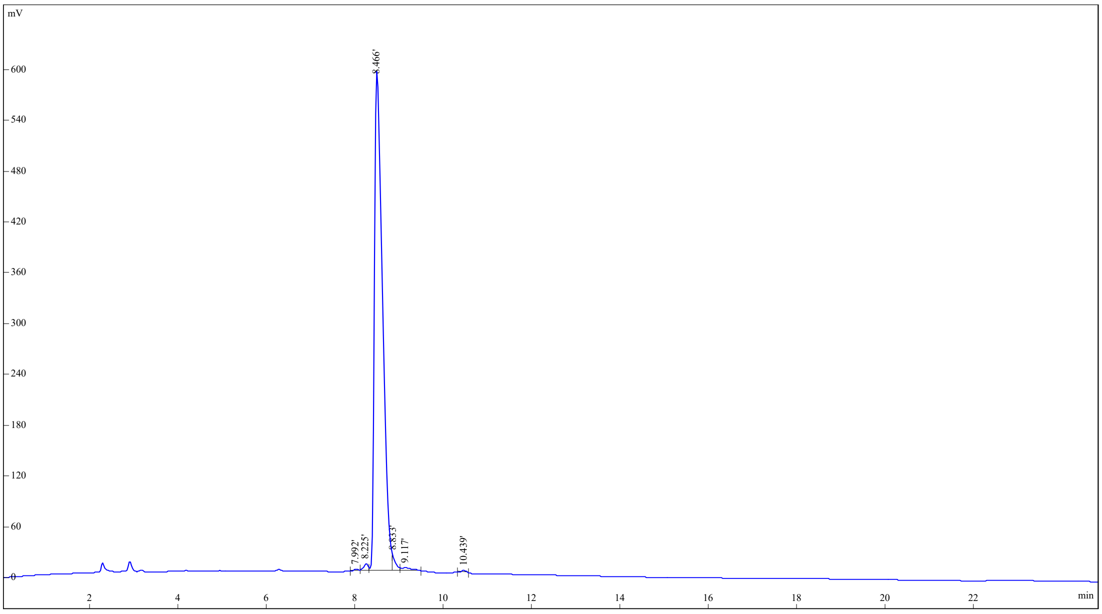

Figure S5. RP-HPLC chromatogram of peptide FRS.

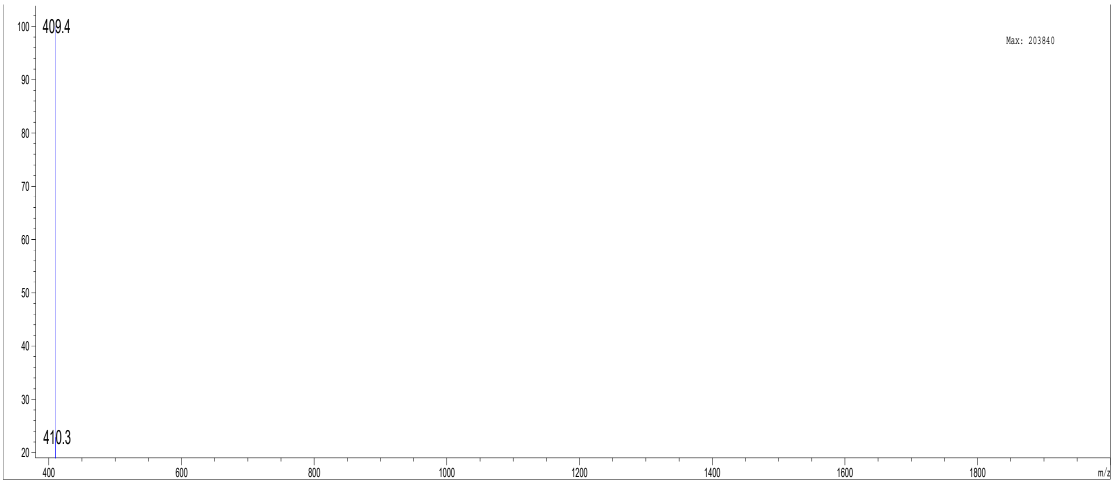

Figure S6. ESI-MS spectrum of peptide FRS.

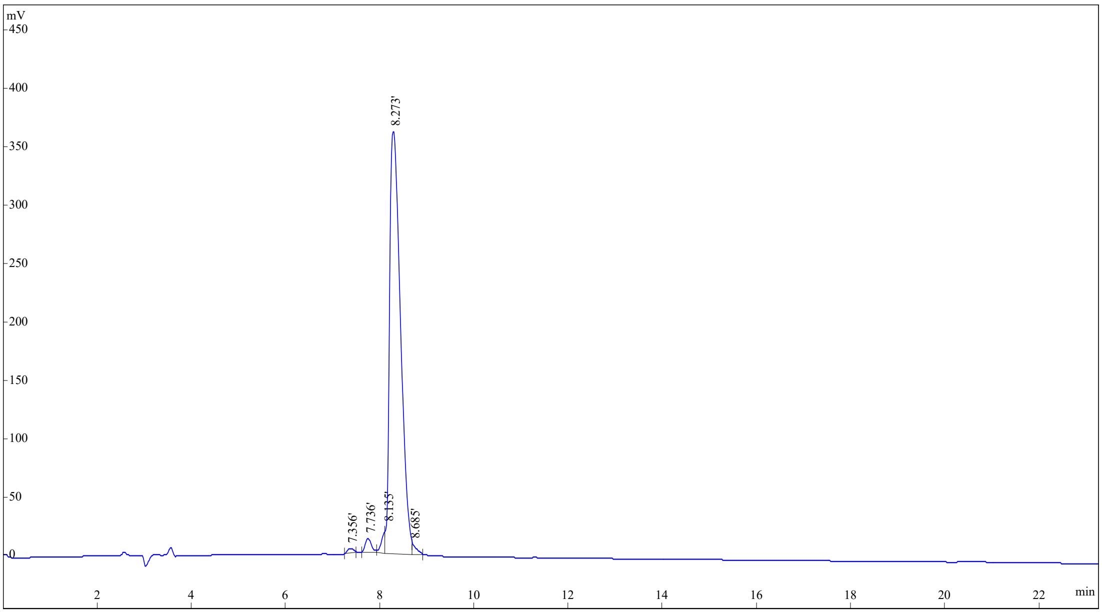

Figure S7. RP-HPLC chromatogram of peptide RRF.

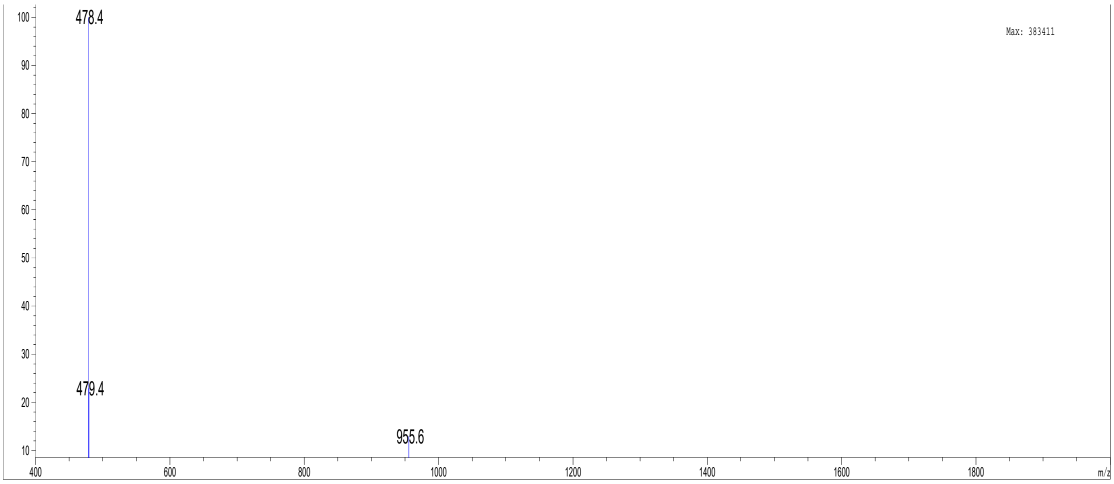

Figure S8. ESI-MS spectrum of peptide RRF.
